# Annotation and profiling of barley *GLYCOGEN SYNTHASE3/Shaggy*-like genes indicated shift in organ-preferential expression

**DOI:** 10.1371/journal.pone.0199364

**Published:** 2018-06-19

**Authors:** Jolanta Groszyk, Yuliya Yanushevska, Andrzej Zielezinski, Anna Nadolska-Orczyk, Wojciech M. Karlowski, Waclaw Orczyk

**Affiliations:** 1 Department of Genetic Engineering, Plant Breeding and Acclimatization Institute–National Research Institute, Radzikow, Blonie, Poland; 2 Department of Computational Biology, Institute of Molecular Biology and Biotechnology, Faculty of Biology, Adam Mickiewicz University, Poznan, Poland; 3 Department of Functional Genomics, Plant Breeding and Acclimatization Institute–National Research Institute, Radzikow, Blonie, Poland; National Taiwan University, TAIWAN

## Abstract

GLYCOGEN SYNTHASE KINASE3/Shaggy-like kinases (GSKs) represent a highly conserved group of proteins found in all eukaryotes. In plants they are encoded by multigene families and integrate signaling of brassinosteroids, auxin and abscisic acid in wide range of physiological and developmental processes with a strong impact on plant responses to environmental and biotic factors. Based on comprehensively studied structures of 10 *Arabidopsis thaliana GSK* genes and encoded proteins we report identification and phylogenetic reconstruction of 7 transcriptionally active *GSK* genes in barley. We re-evaluated annotation of the *GSK* genes in the current barley genome (Hv_IBSC_PGSB_v2) and provided data that a single gene annotated in the previous barley genome ensemble should be retained in the current one. The novel structure of another *GSK*, predicted in Hv_IBSC_PGSB_v2 to encode both GSK and amine oxidase domains, was proposed and experimentally confirmed based on the syntenic region in *Brachypodium distachyon*. The genes were assigned to 4 groups based on their encoded amino acid sequences and protein kinase domains. The analysis confirmed high level of conservation of functional protein domains and motifs among plant GSKs and the identified barley orthologs. Each of the seven identified *HvGSK* genes was expressed indicating semi-constitutive regulation in all tested organs and developmental stages. Regulation patterns of *GSKs* from the indicated groups showed a shift in organ-preferential expression in *A*. *thaliana* and barley illustrating diversification of biological roles of individual *HvGSKs* in different plant species.

## Introduction

GLYCOGEN SYNTHASE KINASE3 (GSK3) represents a highly conserved group of kinases found in all eukaryotes. In plants the GSK3s also designated as GSK3/Shaggy-like kinases (GSK) function as key regulators of diverse physiological and developmental processes with a strong impact on plant responses to biotic and environmental factors. The GSKs are encoded by multigene families. The 10 *A*. *thaliana* genes encoding Shaggy/GSK3-like kinases (AtSK) [1], represent the most comprehensively studied group of plant GSKs. They have been shown to possess diverse functions in the regulation of growth [2–4], responses to environmental and biotic factors [5–8], and development of flowers, stomata, seeds and roots [9–13]. The molecular mechanisms of plant GSKs function is best characterized in brassinosteroid (BR) signaling [14–17] and may overlap with the range of physiological and developmental processes regulated by BRs [18, 19].

The *AtSK* genes are classified into 4 groups based on in their structure, phylogeny [19], sensitivity to AtSK-specific inhibitor bikinin as well as their possible involvement in BR-signaling pathways [15, 20]. *AtSK21*, originally identified as *BRASSINOSTEROID INSENSITIVE2 BIN2* was the first identified and is the best characterized AtSK in *A*. *thaliana* [21, 22]. It phosphorylates BZR1and BES1/BZR2 proteins—two BR-dependent transcription factors (TF) [23]. *ASK21* along with *ASK22* and *ASK23* are assigned to group II of the *AtSK* gene family. All of them were shown to be strongly inhibited by bikinin [15]. The best documented function for this GSK group is involvement in BR signaling. The remaining 7 *AtSKs* belong to groups I, III or IV. *AtSK11*, *AtSK12* and *AtSK13* are assigned to group I. AtSK12 was found to interact and to phosphorylate BZR1and BES1/BZR2 indicating that similar to AtSK21, it acts as a negative regulator of BR signaling [3, 24]. The finding is consistent with inhibition of this set of AtSKs by bikinin [15]. Besides involvement in BR-dependent signaling there are reports linking some of the group I AtSKs to physiological response to environmental factors [5]. Stress-activated AtSK11 was reported to phosphorylate glucose-6-phosphate dehydrogenase (G6PD) and participate in cell protection against oxidative stress [5]. This finding shows that another phosphorylation targets (besides the BZR1and BES1/BZR2) may lead to alternative non-BRs related functional paths. The genes *AtSK31* and *AtSK32* were assigned to group III but only one of them (*AtSK31*), which is moderately inhibited by bikinin [15], was shown to participate in BR signaling [14].

The detailed functions of group IV *AtSK41* and *AtSK42* are not known. The two features of AtSK42, i.e., very weak inhibition by bikinin and different structure of the ATP-binding pocket, relative to other ASKs, argue against its involvement in BR signaling [15]. *MSK4*, the *Medicago sativa* ortholog of *AtSK41was* shown to regulate salt tolerance by adjusting carbohydrate metabolism in response to environmental stress, which might indicate the possible function of group IV GSKs [7, 25]. At least seven of ten *AtSKs* (*AtSK11*, *12*, *13*, *21*, *22*, *23* and *31*) were reported to function as BR regulators but only *AtSK21*, *AtSK22*, *AtSK23* and *AtSK31* were fully analyzed in biochemical and genetic studies [2, 14, 17, 18, 24, 26]. Different *AtSK* members can have redundant, but not fully overlapping functions. Transcripts of *AtSKs* are present in all major organs and developmental stages [27]. The genes show semi-constitutive expression pattern with certain level of organ or developmental stage dependent regulation. Relatively strongest expression of all *AtSKs*, particularly group III *AtSK31* and *AtSK32*, was found in inflorescence stems, flower buds and open flowers, implying they may be involved in generative development [9, 27–29]. The lowest transcript level was found for group IV genes: *AtSK411* and *AtSK42* [27].

Here, we report identification and phylogenetic evaluation of 7 transcriptionally active *GSK* genes in barley. Specifically, we: (1) assign *GSK* genes to four groups based on their evolutionary relationships and expression patterns with known *AtSK* genes, (2) analyze the gene structure and composition of *GSK* family members assigned to 4 these groups, (3) identify shifts in tissue-preferential expression that may relate to functional diversification in barley and (4) re-evaluate annotation of GSK genes in the most recent barley genome release (Hv_IBSC_PGSB_v2).

## Materials and methods

### Plant material and growth conditions

The barley (*Hordeum vulgare* L) cultivar Golden Promise was used as a source of plant material in all experiments. After 72 hours of imbibition barley kernels were planted in pots (14 cm diameter) filled with peat substrate mixed with sand in a 10:3 v/v ratio. The seedlings were cultivated in a growth chamber with a 16 h photoperiod, at 22°C in the day and 18°C at night. The relative humidity was in the range 60–80%, and the light intensity was 150 μM⋅s^-1^m^-2^. Plants were irrigated twice a week and fertilized once a week with the multicomponent soil fertilizer Florovit (http://florovit.pl/) according to the manufacturer’s instructions. Plant samples for expression profiling were collected from leaves and roots of 5-days old seedlings, the leaves and roots of 14-days old seedlings, stem with developing ear, pre-meiotic ear, meiotic ear, ear 0 days after pollination (DAP), ear at 7 DAP and ear at 14 DAP.

### Sequence data retrieval and identification of GSK3s family members in barley

Genome annotation layers for *H*. *vulgare* (Hv_IBSC_PGSB_v2) and *A*. *thaliana* (TAIR10) and *Brachypodium distachyon* (v. 1) were retrieved from Ensembl Plants [30]. In order to minimize the number of mis- or non-annotated GSK proteins (false negative predictions), the identification of GSK family members was simultaneously carried out on three layers of evidence: (i) amino acid sequence similarity assessed by BLAST (e-value ≤ 10^−20^) [31] as well as ORCAN metaserver that includes four orthology identification methods [32]; (ii) tblastn-based DNA level similarity searches with genomic sequence and e-value cut-off at 10^−20^, in order to check whether some potential GSK members were missed during gene annotations; additionally, NCBI’s dbESTs database was searched for GSK genes from barley to additionally improve the sampling of the lineages; (iii) protein domain level, where kinase domains and ATP binding sites were identified in the set of barley proteins using PfamScan provided by Pfam database [33] and PS_SCAN provided by PROSITE database [34]. Both domain identification programs were run with default parameters (e-value ≤ 10^−5^).

### Phylogenetic reconstruction

The kinase domain sequences in selected homologous GSK proteins in barley and *A*. *thaliana* were retrieved (using Pfam annotations—Pfam AC: PF00069) and extracted from their full-length parental sequences. The alignments of the kinase domain amino acid sequences were conducted using the MAFFT program [35] Maximum likelihood (ML) phylogenetic analyses were conducted using MEGA7 [36] with the JTT model of evolution with bootstrap support calculated over 1000 replications. Sequence from the moss *Physcomitrella patens* (UniProt Acc: A9S6L2) was set as an outgroup.

### Nucleic acid isolation, reverse transcription and transcript quantification

The total RNA was extracted from plant samples using TRI Reagent (Sigma Aldrich, Germany). RNA concentration and A260/280 ratio (always higher than 1.8) were measured using a NanoDrop spectrophotometer (NanoDrop Technologies, USA). The RNA quality was further determined by agarose electrophoresis. Isolated total RNA was treated with 2 U of DNase (RNase-free, Roche, USA) and 2 U of Protector RNase inhibitor followed by DNase inactivation according to the manufacturer’s protocol. Genomic DNA impurities were checked by PCR with primers qAct1 and qAct2a (S1 Table) specific to the β-actin gene, and 100 ng of DNase treated RNA as a template. Complete removal of gDNA was confirmed by lack of detectable amplicon after 36 cycles of amplification. Two micrograms of RNA (DNase-treated with undetectable gDNA impurities) were used as a template for the reverse transcription reaction with oligo d(T) primers using RevertAid First Strand cDNA Synthesis Kit (Thermo Scientific, USA). The obtained cDNA was diluted twentyfold and used directly as a template for quantitative PCR (qPCR). The standard qPCR reaction mix was composed of 2.2 μL of 5x HOT FIREPol EvaGreen qPCR Mix Plus (ROX) (Solis Biodyne, Estonia), 0.25 μL of primer F (10 μM), 0.25 μL of primer R (10 μM) (S1 Table), 3 μL of cDNA and water to 11 μL. The reaction was performed in a Rotor-Gene 6000 model 5-plex thermocycler (Corbett, Australia). The efficiencies of amplification for all primer pairs were in the range 0.9 to 1.00 and R^2^ 0.99875–1.00000. The specificity of amplification was verified by melting curve analysis. Template concentrations ranging from 10^4^ to 10^8^ copies of analyzed amplicon and 10^4^−10^8^ copies of the reference gene ADP-ribosylation factor (ADP RF) AJ508228 per reaction were used as the standards for qPCR [37–39]. Threshold line, Ct values, standard curves and relative quantifications were determined using the proprietary Rotor-Gene 6000 software v 1.7. The results of real-time PCR experiments represent at least three biological replicates with three technical repetitions each. The whole procedure of RNA isolation, reverse transcription, qPCR conditions and data analysis met the MIQE criteria outlined by [40]

### Statistical analyses

Statistical analysis was done using Statistica 13.0 software.

## Results

### Barley genome encodes seven GSK genes

**Fig 1:**
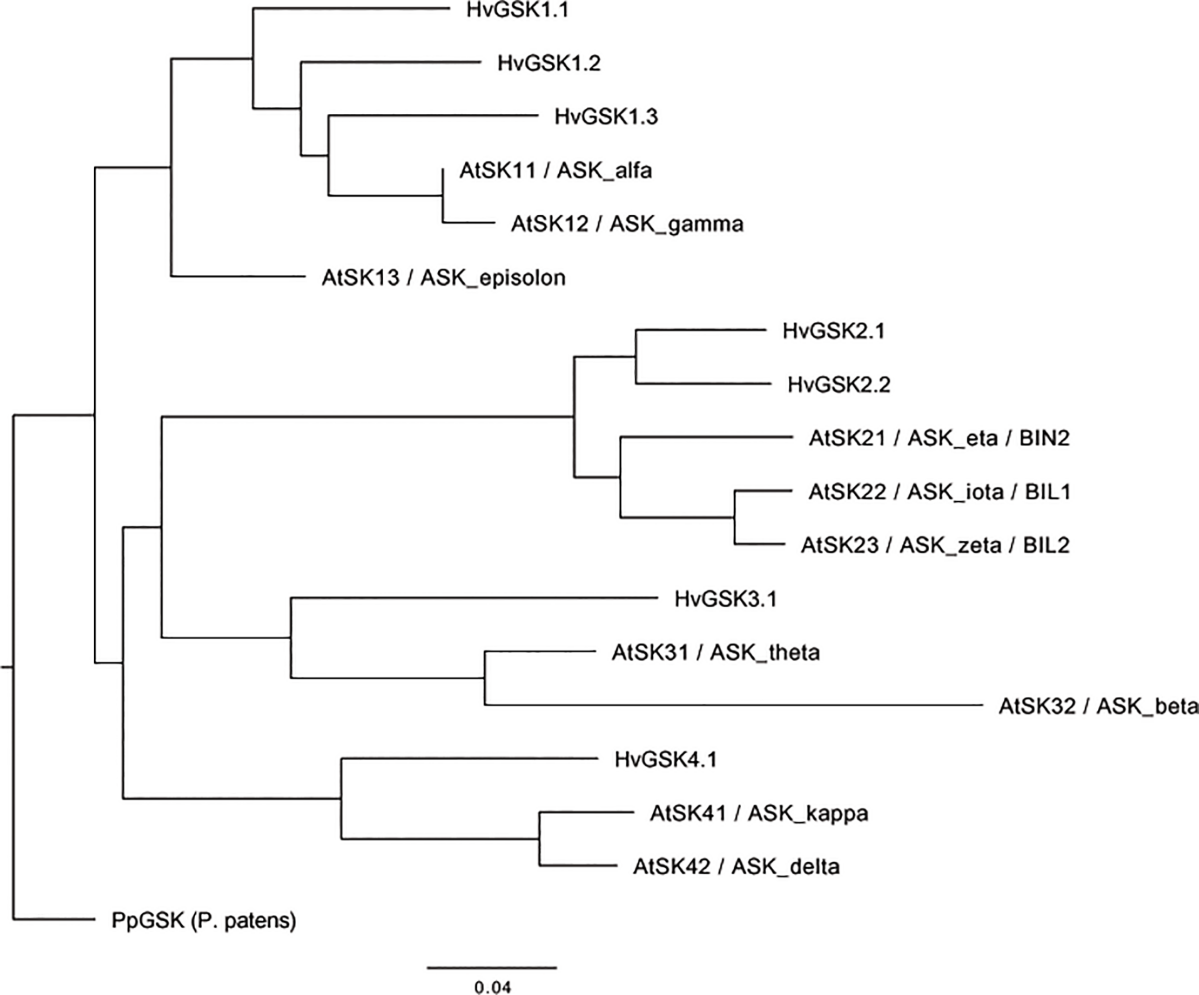
Phylogenic tree of *GSK* genes of *Arabidopsis thaliana* and *Hordeum vulgare* used for classification of barley GSK family members. The phylogeny was reconstructed based on amino acid sequence of kinase domains using maximum likelihood algorithm. GSK protein from *Physcomitrella patens* (PpGSK) was used as an outgroup.

Using three independent computational approaches (for details see Materials and Methods), we have verified genome annotation of barley and confirmed presence of six genes in the most recent annotation (Hv_IBSC_PGSB_v2; http://plants.ensembl.org/Hordeum_vulgare/Info/Index), and one from previous release (Hordeum_vulgare.ASM32608v1) that was removed from current gene set. The selected genes encode proteins with high similarity to the AtSKs (Fig 1, Table 1, S2 Table and S3 Table): HORVU3Hr1G034440 located on chromosome 3H (chr3H), HORVU5Hr1G117030 on chr5H, HORVU1Hr1G016490 on chr1H, HORVU3Hr1G026020 on chr3H, HORVU1Hr1G048580.8 on chr1H and HORVU5Hr1G119790 on chr5H. Based on phylogenetic analysis of amino acid sequence of kinase domains, GSK family members were allocated to four clades (Fig 1; S3 Table). In compliance with this classification the genes were designated as *HvGSKs* with numbers indicating the corresponding group (Table 1). The first three genes: HORVU3Hr1G034440, HORVU5Hr1G117030 and HORVU1Hr1G016490 were assigned to group I and labelled as *HvGSK1*.*1*, *HvGSK1*.*2* and *HvGSK1*.*3*, respectively. The genes have 8, 9 and 15 splice variants and most of them encode a protein with a kinase domain (PS50011), serine/threonine-protein kinase active site (PS00108) and protein kinase ATP binding site (PS00107). The gene HORVU3Hr1G026020 was classified to group II and assigned as *HvGSK2*.*1*. The gene has 19 splice variants and 16 of them contain a protein kinase domain (PS50011), serine/threonine-protein kinase active site (PS00108) and protein kinase ATP binding site (PS00107). The gene HORVU1Hr1G048580 with 22 transcripts was assigned to group III and designated as *HvGSK3*.*1*. All splice variants including a truncated HORVU1Hr1G048580.22 gene contain an intact protein kinase domain typical for GSKs. The HORVU5Hr1G119790 gene was placed in group IV and named as *HvGSK4*.*1*. The predicted gene has a corresponding set of 34 transcripts that can be divided into 2 distinct classes, each characterized by different types of encoded peptide domains (see later: Re-annotation of barley GSK genes) (Table 1).

**Table 1:** *GSK* orthologs in barley and *Arabidopsis thaliana*.

Group	Gene	Ensembl Plants *Ensemble Plants Archive	Barley chromosome	NCBI	Comment	*A*. *thaliana* orthologs
I	*HvGSK1*.*1*	HORVU3Hr1G034440.2	chr3H	AK251287.1	The transcripts: AK251287.1 (1836 bp) and HORVU3Hr1G034440.2 (2019 bp) overlap and encode identical 406 aa peptides.	*AtSK11* *AtSK12* *AtSK13*
	*HvGSK1*.*2*	HORVU5Hr1G117030.1	chr5H	AK368391.1	The transcripts: AK368391.1 (1698 bp) and HORVU5Hr1G117030.1 (1840 bp) overlap and encode identical 408 aa peptides.	
	*HvGSK1*.*3*	HORVU1Hr1G016490.9	chr1H	none	The HORVU1Hr1G016490.9 transcript (1845 bp) encodes 410 aa peptide. There is no cDNA clone similar to HORVU1Hr1G016490.9 in NCBI database.	
II	*HvGSK2*.*1*	HORVU3Hr1G026020.1	chr3H	AK364823.1 AK360132.1	The transcripts: AK360132.1 (1515 bp), AK364823.1 (1734 bp) and HORVU3Hr1G026020.1 (3184 bp) overlap and encode identical 400 aa peptides.	*AtSK21 AtSK22 AtSK23*
	*HvGSK2*.*2*	*MLOC_68311.2	*chr1H	none	MLOC_68311.2 is not included in the current assembly of barley genome although it was present in the previous barley genome release. See further explanation in the manuscript.	
III	*HvGSK3*.*1*	HORVU1Hr1G048580.7	chr1H	AK362547.1	The transcripts: AK362547.1 (1906 bp) and HORVU1Hr1G048580.7 (2169 bp) overlap and encode identical 468 aa peptides.	*AtSK31 AtSK32*
IV	*HvGSK4*.*1*	HORVU5Hr1G119790.18	chr5H	AK358344.1 AK360683.1	The transcripts: AK358344.1 (1777 bp), AK360683.1 (1813 bp) and HORVU5Hr1G119790.18 (1528 bp) overlap and encode highly similar amino acid sequences.	*AtSK41 AtSK42*

*—ASM32608v1; Ensemble Plants Archive

Catalytic domains of HvGSKs show on average very high sequence resemblance (similarity: 93% ± 3%, identity: 84% ± 5%) to corresponding domains of AtSKs. Alignment of the GSK protein sequences from *A*. *thaliana*, barley and *P*. *patens* allowed identification of the ATP-binding motif located within the boundaries of the kinase domain (Pfam AC: PF00069) (S3 Table). Additionally, all barley GSKs have conserved TREE sequence motif typical for AtSK21 protein (S3 Table) [22]. The highly-conserved plant GSKs amino acid motifs: CDFGSAK and GEPNISYICSR [25] are also present in all barley GSKs except HvGSK2.1, where first serine (S) residue in the latter motif is substituted by alanine (A). Tyrosine (Y) present in this motif corresponds to T200 in AtSK21 and T216 in human/mouse GSK3α and GSKβ [41]. The SIDIV motif typical for group II AtSKs [25] is present in both HvGSK2.1 and HvGSK2.2 that were also assigned to group II (S3 Table). The MEYV and LEYV motifs, with M115 or L115, Y117 and V118 residues were reported to be important for bikinin docking within the ATP-binding pocket [15]. The MEYV motif present in AtSK21, AtSK22 and AtSK23 is also found in HvGSK2.1 and HvGSK2.2. The LEYV motif was present in GSKs from group I, III and IV in *A*. *thaliana* and barley. A single F117 to Y115 substitution in LEFV found in AtSK41 and AtSK42 was also present in HvGSK4.1 assigned to group IV [15] (S3 Table).

### Re-annotation of barely GSK genes

The previous release of barley genome assembly (ASM32608v1; *Ensemble Plants* Archive) contained gene MLOC_68311.2 located on chr1H, which is not present in the current release (Hv_IBSC_PGSB_v2). Full-length protein sequence of MLOC_68311.2 shows highest sequence identity (86.8%) and similarity (90.4%) to HvGSK2.1. Moreover, transcript sequences of both genes show also highest (70.8%) similarity. Additionally, phylogenetic analysis groups both genes as paralogs (Fig 1) and both sequences, similarly to Arabidopsis AtSK21, AtSK22 and AtSK23, contain conserved SIDIW motif (S3 Table), which seems to be a unique feature of clade II GSKs. Although MLOC_68311.2 does not have any corresponding cDNA clones in the GenBank database, the presence of over 82 barley ESTs (NCBI dbEST database) covering the whole 1896 bp transcript with over 99% identity suggest that the gene is actively transcribed. Therefore, we have included the MLOC_68311.2 gene in group II and designated it as *HvGSK2*.*2* (Table 1).

**Fig 2:**
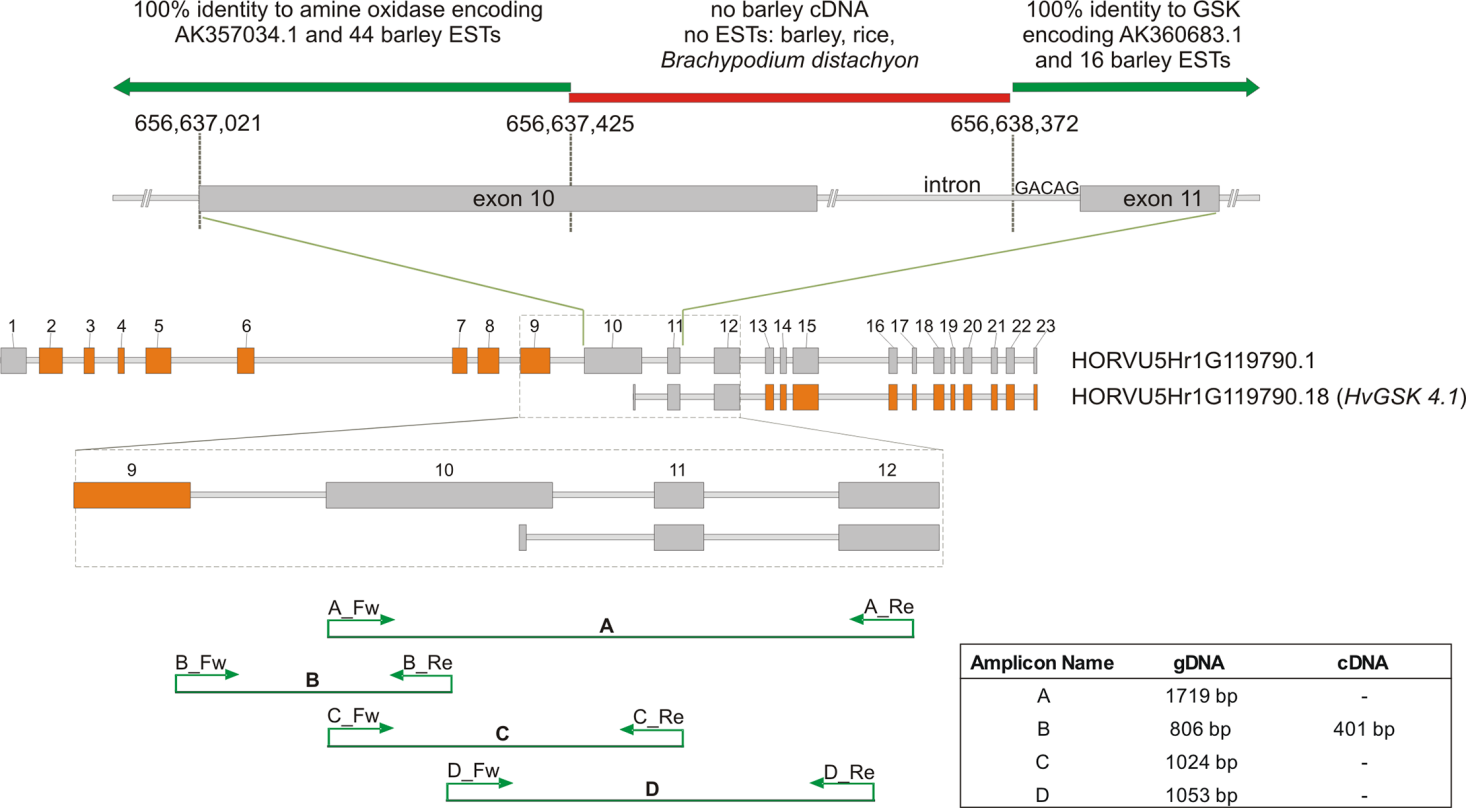
Schematic structure of HORVU5Hr1G119790.1 transcript predicted to encode amine oxidase and Glycogen Synthase Kinase (GSK) and HORVU5Hr1G119790.18 transcript annotated as *HvGSK4*.*1*. Enlarged exon10-exon11 fragment with non-transcribed junction between amine oxidase and GSK encoding regions. Indicated are nucleotide coordinates from the current barley genome Hv_IBSC_PGSB_v2.Enlarged exon9-exon12 region of HORVU5Hr1G119790.1 and HORVU5Hr1G119790.18 (*HvGSK4*.*1*) transcripts. Indicated are annealing sites of primers and results of amplification using barley gDNA or cDNA as template. Nucleotide alignment of amplicon A and the corresponding fragment of HORVU5Hr1G119790 gene are presented as S4 Table.

In current genome annotation the HORVU5Hr1G119790 gene shows very complex exon-intron structure and a large set of 34 splicing variants that encode proteins with two distinct sets of functional domains. Six transcripts encode peptides with FAD/NAD(P)-binding site and amine oxidase domain. One transcript encodes a very short protein lacking any known domains. The remaining 17 transcripts representing the second class, encode peptides with protein kinase domain, serine/threonine-protein kinase active site and protein kinase ATP binding site arranged in a similar mode as in other GSKs. Both main groups of predicted transcripts are supported in the NCBI nucleotide database by cDNAs and ESTs. The first 9 exons and the 404 bp 5’-fragment of the exon 10 of the HORVU5Hr1G119790.1 transcript encode amine oxidase. Their nucleotide sequences are identical to AK357034.1, AK363738.1 and 44 ESTs. The remaining exons (from 11 to 23) of the same HORVU5Hr1G119790 gene encode protein with GSK domains and show 100% similarity to AK360683.1, AK358344.1 and 16 ESTs. The short 3’-end fragment of the exon 10 has no similarity to any known barley cDNA or EST, suggesting that this region of genomic DNA is not transcribed (Fig 2). Since none of the *A*. *thaliana GSK* genes have such complex structure as the HORVU5Hr1G119790 we decided to verify the assembly of this particular region of barley genome to check whether it encodes a single, predicted transcript. PCR amplification with primers (S1 Table) anchored to the exons 9, 10, 11 and 12 of the HORVU5Hr1G119790.1 putative transcript using cDNA or genomic DNA of cv. Morex as the templates gave amplicons of expected sizes and nucleotide sequences or failed to amplify detectable signal (Fig 2). PCR reactions with primers flanking exons 10, 11 and 12 gave products only using gDNA as a template. The lengths of the fragments and their nucleotide sequences were as predicted for the HORVU5Hr1G119790 gene (Fig 2, S4 Table). The same primers used for reactions with barley cDNA as a template failed to amplify any products (Fig 2). These results confirm the current assembly of this region indicating that both parts of the HORVU5Hr1G119790 gene i.e. encoding amine oxidase and GSK are located in barley genome as predicted. However, no amplification of exons 10, 11, and 12 using cDNA as a template (Fig 2) implies that there is no continuous single-molecule transcript as proposed for the HORVU5Hr1G119790.1 gene. Contrary, the results point to the presence of two separate transcripts: one encoding an amine oxidase and other a GSK protein. The 947 bp fragment, positioned from 656,637,425 to 656,638,372 of chromosome 5 and spanning amine oxidase and GSK regions, represents non-transcribed fragment of the genome (Fig 2). In conclusion, we propose that the region of the barley chromosome 5 (chr5H) starting from 656,638,372 to 656,642,630 represents a separate transcription unit encoding a group IV GSK-like kinase. Hence the gene was designated as *HvGSK4*.*1*.

### Omnipresent expression of GSK genes across organs and developmental stages

**Fig 3:**
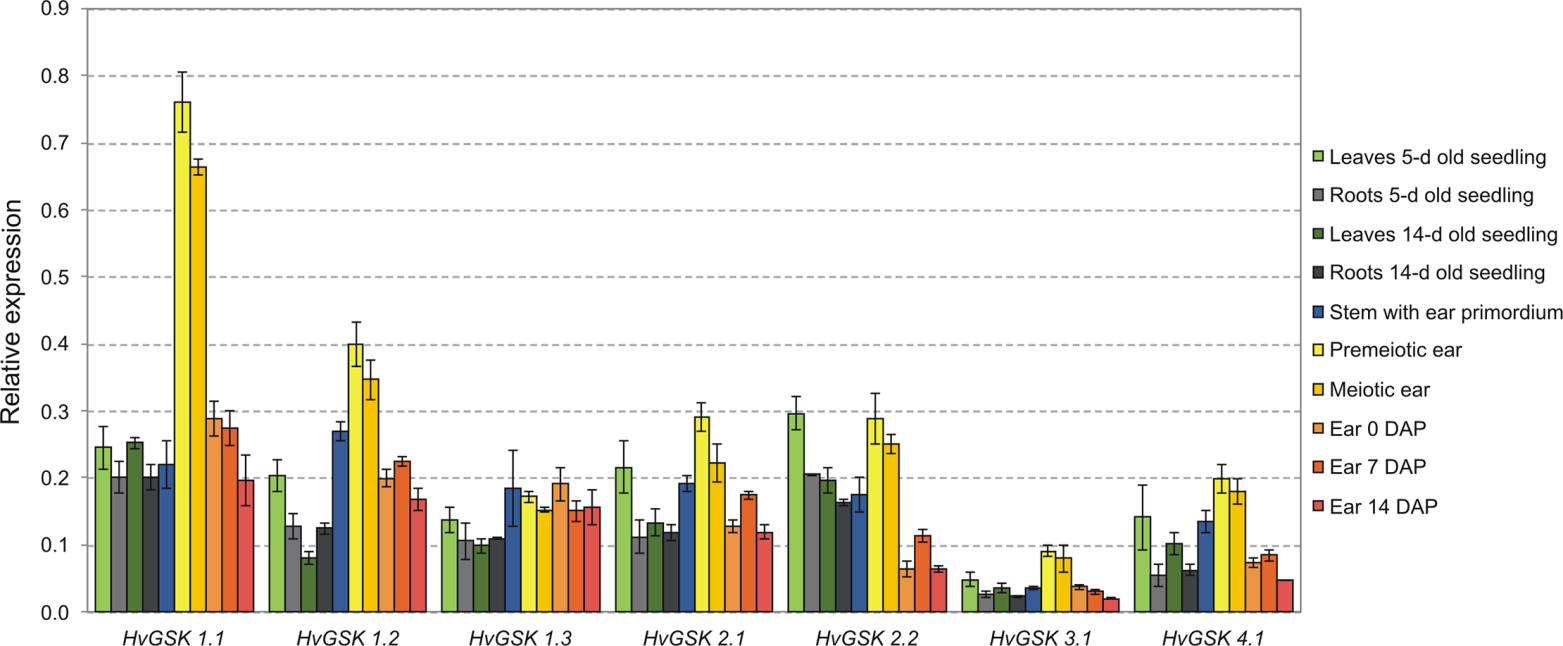
Relative expression profile of the 7 *HvGSK* genes in selected organs and developmental stages of barley plants. The results represent the mean values and SD of a ratio of the studied gene transcript to the transcript of ADP-ribosylation factor used as an internal reference.

Transcript levels of *HvGSK* genes were tested in leaves and roots (from 5 and 14 days old seedlings), in stems with ear primordia and in ears collected from 5 developmental stages: premeiotic, meiotic and 0, 7 and 14 days after pollination (DAP). Transcripts of all 7 barley *GSK* genes were present in each of tested organs and developmental stages indicating that expression of members of this gene family is semi-constitutive. Regulation of *HvGSKs* in leaves and roots of 5 and 14 days old seedlings showed common pattern: the two genes, *HvGSK1*.*1* form group I and *HvGSK2*.*2* form group II had the highest expression. In leaves of 5-d old seedlings the strongest signal showed *HvGSK1*.*1* (0.25) and *HvGSK1*.*2* (0.20) from group I, and *HvGSK2*.*1* (0.22), *HvGSK2*.*2* (0.30) from group II. Similarly the strongest expression in roots of 5-d old seedlings showed *HvGSK1*.*1* (0.20) from group I and *HvGSK2*.*2* (0.20) from group II (Fig 3). In leaves and roots of 14-d old seedlings the highest expression was detected for *HvGSK1*.*1* from group I (0.25 and 0.20 in leaves and roots, respectively) and *HvGSK2*.*2* from group II (0.20 and 0.16 in leaves and roots, respectively). The strongest expression in stems with ear primordia showed genes from group I: *HvGSK1*.*2* (0.27) and *HvGSK1*.*1* (0.22) (Fig 3). In vegetative organs the levels of genes’ expression were mostly not correlated with each other. Out of 21 gene to gene combinations only 4 pairs showed significant correlation in leaves and stem and 6 pairs in roots (Table 2).

**Table 2:** Pearson correlation coefficients of relative expression of *HvGSK* in selected organs and developmental stages. Significant correlations (p <0.05) are indicated by asterisk (*).

A	**Vegetative: above ground, leaves and stem.**						
	*GSK 1*.*1*	*GSK 1*.*2*	*GSK 1*.*3*	*GSK 2*.*1*	*GSK 2*.*2*	*GSK 3*.*1*	*GSK 4*.*1*
*GSK 1*.*1*		-0.321	0.124	0.157	0.392	0.299	0.087
*GSK 1*.*2*	-0.321		0.785*	0.735*	0.053	0.366	0.524
*GSK 1*.*3*	0.124	0.785*		0.529	-0.027	0.250	0.330
*GSK 2*.*1*	0.157	0.735*	0.529		0.586	0.821*	0.679*
*GSK 2*.*2*	0.392	0.053	-0.027	0.586		0.611	0.526
*GSK 3*.*1*	0.299	0.366	0.250	0.821*	0.611		0.256
*GSK 4*.*1*	0.087	0.524	0.330	0.679*	0.526	0.256	
B	**Vegetative: roots.**						
	*GSK 1*.*1*	*GSK 1*.*2*	*GSK 1*.*3*	*GSK 2*.*1*	*GSK 2*.*2*	*GSK 3*.*1*	*GSK 4*.*1*
*GSK 1*.*1*		0.908*	0.806	0.962*	-0.178	0.581	0.712
*GSK 1*.*2*	0.908*		0.828*	0.920*	0.122	0.853*	0.653
*GSK 1*.*3*	0.806	0.828*		0.925*	-0.350	0.555	0.764
*GSK 2*.*1*	0.962*	0.920*	0.925*		-0.270	0.588	0.801
*GSK 2*.*2*	-0.178	0.122	-0.350	-0.270		0.574	-0.351
*GSK 3*.*1*	0.581	0.853*	0.555	0.588	0.574		0.306
*GSK 4*.*1*	0.712	0.653	0.764	0.801	-0.351	0.306	
C	**Generative organs: premeiotic and meiotic ears, kernels 0, 7 and 14 days after pollination.**						
	*GSK 1*.*1*	*GSK 1*.*2*	*GSK 1*.*3*	*GSK 2*.*1*	*GSK 2*.*2*	*GSK 3*.*1*	*GSK 4*.*1*
*GSK 1*.*1*		0.992*	0.001	0.941*	0.981*	0.997*	0.995*
*GSK 1*.*2*	0.992*		-0.052	0.975*	0.991*	0.983*	0.996*
*GSK 1*.*3*	0.001	-0.052		-0.099	-0.173	0.074	-0.060
*GSK 2*.*1*	0.941*	0.975*	-0.099		0.968*	0.923*	0.952*
*GSK 2*.*2*	0.981*	0.991*	-0.173	0.968*		0.963*	0.989*
*GSK 3*.*1*	0.997*	0.983*	0.074	0.923*	0.963*		0.989*
*GSK 4*.*1*	0.995*	0.996*	-0.060	0.952*	0.989*	0.989*	

Expression profiles in generative organs, i.e. selected stages of ear development, showed several distinct features when compared to expression in vegetative tissues: leaves, roots and stems. The most characteristic in premeiotic and meiotic ears was a strong expression of *HvGSK1*.*1* (0.76 and 0.66 in pre- and meiotic ears respectively) and *HvGSK1*.*2* (0.40 and 0.35 in pre- and meiotic ears, respectively) both belonging to GSK group I. Expressions of the remaining genes were lower and ranged from 0.08 to 0.29 (Fig 3). Expression of genes from group I in ears at 0, 7 and 14 DAP (ranging from 0.15 to 0.29) dominated over the genes from groups II, III and IV (ranging from 0.02 to 0.17). The lowest level of transcript in all tested samples, ranging from 0.02 to 0.09, showed *HvGSK3*.*1* (Fig 3). Relative expression in premeiotic ears was strongly diversified but 15 gene pairs out of 21 showed significant and strong positive correlation. It is worth to note that *HvGSK1*.*3* was a single gene non-correlating with any of the other *HvGSKs* (Table 2). Despite big differences in detected transcript levels, the patterns of expression in tested organs showed some common features: they were higher in young generative organs i.e., premeiotic and meiotic ears than in vegetative organs and older ears (Fig 3).

## Discussion

Our analysis confirmed high level of conservation of functional protein domains and motifs among plant GSK proteins. It is highly consistent with previous reports on protein and gene structure [18, 19, 42–44]. Based on sequence features, the 7 barley *GSK* genes were assigned to the 4 groups which correspond to the 4 clades of the *AtGSK* genes originally proposed by Jonak and Hirt [19].

In concordance with data for other plant species [44] the group I in barley includes 3 genes i.e.: *HvGSK1*.*1*, *HvGSK1*.*2* and *HvGSK1*.*3*. Group II, in contrast, shows variable number of genes (from 2 in *Sorghum bicolor* to 5 *Zea mays*) that most probably reflect different number of whole genome duplication events [44]. The current assembly of barley genome contains a single gene HORVU3Hr1G026020 (*HvGSK2*.*1*) located on chr3H. The previous release of barley genome (ASM32608v1) contained another gene MLOC_68311.2 located on chr1H. This gene however, was not included in the recent barley genome annotation (Hv_IBSC_PGSB_v2). Our results confirmed the presence of MLOC_68311.2 cDNA in barley transcriptome and showed that its expression pattern is similar to *HvGSK2*.*1*. Therefore, we postulate that both genes i.e. the *HvGSK2*.*1* (HORVU3Hr1G026020) and the *HvGSK2*.*2* (MLOC_68311.2) are actively transcribed in barley genome and constitute the group II of barley *GSKs*. The only one gene (*HvGSK3*.*1*) classified to group III in barley, corresponds well with quantity of genes in most monocot species. Only *A*. *thaliana* and *S*. *bicolor* have 2 *GSK* genes assigned in this group [44]. Similar situation is present in group IV, where all monocot species, including described in this study *HvGSK4*.*1* (HORVU5Hr1G119790), contain only one gene, whereas *A*. *thaliana* has 2 *AtSKs*. One of the annotated genes i.e. HORVU5Hr1G119790 shows unexpectedly high number of 34 transcripts encoding amine oxidase and GSK-type proteins. This would be a first case of known *GSK* genes encoding proteins with two types of domains i.e. amine oxidase and GSK. However, a similar arrangement of two neighboring genes: the one encoding amine oxidase and the other one GSK can be found in *B*. *distachyon* genome (S1 Fig). The first *B*. *distachyon* gene BRADI1G02150 is located on chromosome 1 at positions: 1,460,089–1,464,617 and encodes protein with FAD/NAD(P) binding sites and amine oxidase domain. The second gene BRADI1G02160 (a putative *BdGSK4*.*1*) is located 2328 bp down-stream and encodes a peptide with domains typical for GSK: protein kinase domain, Ser/Thr protein kinase active site and protein kinase ATP binding site. The expected synteny of this region between *B*. *distachyon* and barley and lack of any known *GSK* genes encoding both amine oxidase and GSK domains prompted us to verify the genomic assembly and predicted transcripts of the HORVU5Hr1G119790 gene. The results validated the current genomic arrangement (Hv_IBSC_PGSB_v2) of the two neighboring regions of the HORVU5Hr1G119790 gene. The results also confirmed the local genomic synteny between *B*. *distachyon* and *H*. *vulgare*. However, presented results indicate that the region spanning both parts of HORVU5Hr1G119790.1 is not transcribed. The former fully agrees with the lack in databases of cDNAs or ESTs corresponding to this region. In conclusion, the HORVU5Hr1G119790.1 transcript does not exist as a single molecule as it is reported in the current assembly of barley genome. Instead, the region assigned as HORVU5Hr1G119790 gives two independent transcripts, where one of them encodes the barley GSK protein (HvGSK4.1).

Each of the seven identified *HvGSK* genes was expressed in all tested organs and developmental stages. The results are compatible with expression of *GSK* genes reported in other plant species. In *A*. *thaliana* the transcripts of *AtSK* genes were detected in all tested tissues and showed semi-constitutive expression at the organ level [27]. This type of regulation very well corresponds to the known participation of these genes in wide range of biological and developmental processes. In barley the highest expression level was observed in group I: *HvGSK1*.*1* and *HvGSK1*.*2* with the peak in early developmental stages of generative organs: premeiotic, meiotic and 0DAP ears. This agrees with the strong expression of *AtSK11* and *AtSK12* in flowers and with involvement of these genes in flower development [28]. *In situ* hybridization indicated that both *ATSK11* and *AtSK12* regulated number of domains in flower meristem [28] but not in developing embryos [25]. According to Qi et al. [44] flower-preferential expression and roles in floral functions of *AtSK11* and *AtSK12* may be evolutionarily conserved in most angiosperms. The conclusion is further supported by reported here strong up-regulation of *HvGSK1*.*1* and *HvGSK1*.*2* in premeiotic and meiotic ears. Expression of the group II *GSKs* was lower compared to the genes from group I, but regulation patterns in the both groups remained similar. Expression of the *HvGSK2*.*1* and the *HvGSK2*.*2* were enhanced in leaves of 5-d old seedlings and in premeiotic and meiotic ears. This concurs with transcriptional activity of the group II *AtSKs* in generative organs, developing seeds and embryos. Transcripts of *AtSK21*, *AtSK22* and *AtSK23* were found in epidermal and subepidermal layers in early stages of seed development. Localization of the transcripts within generative organs was distinct and specific for each gene. The *AtSK23* transcript was detectable in the whole embryo, while *AtSK21* could be only localized in the suspensor cells [25]. The highest levels of *AtGSK1* (the gene is identical to *AtSK22*) were in flowers and siliques while it was quite low in leaf and root tissues [45].

Regulation patterns of *GSKs* from the 4 groups illustrate a shift in organ-preferential expression observed in *A*. *thaliana* and barley. Group I in barley (*HvGSK1*.*1* and *HvGSK1*.*2*) and group III in *A*. *thaliana* (*AtSK31*) showed the highest expression in young generative organs. This shift in expression might reflect different roles of these two GSK groups in regulation of flower development in dicot *A*. *thaliana* and monocot barely [4, 28, 46]. The highest expression of barley group I genes in premeiotic and meiotic ears and *A*. *thaliana* group III in inflorescence and flower buds [27, 47, 48] confirms their role in generative development. In *A*. *thaliana* the lowest expression was reported for group IV (*AtSK41*, *AtSK42*) [27] while the lowest expression in barley was in group III (*HvGSK3*.*1*). Considering this shift of expression levels it may be hypothesized, that group IV of GSKs, preferentially involved in osmotic stress [27] and carbohydrate metabolism [7], become more biologically important in barley, growing in generally dryer conditions, than in *A*. *thaliana*. Observed in barley and *A*. *thaliana* shifts in organ-preferential expression indicate that biological roles of individual *HvGSKs* might be different compared to other species.

## Supporting information

S1 Fig Schematic structure of genomic region of *Brachypodium distachyon* chromosome1 showing two neighboring genes BRADI1G02150.1 and BRADI1G02160.1. (TIF)

S1 Table List of primers and reaction conditions used in this study. (DOCX)

S2 Table List of GSK-encoding genes in *Arabidopsis thaliana* and barley. (XLS)

S3 Table Amino acid alignment of GSK-encoded proteins in *Arabidopsis thaliana*, barley and *Physcomitrella patens*. Boundaries of the kinase domain (Pfam AC: PF00069) are indicated by black arrows and framed in black. Alignment includes one protein isoform per gene (isoform containing kinase domain with the highest Pfam score).
(DOCX)

S4 Table Nucleotide alignment of amplicon A and the corresponding fragment of HORVU5Hr1G119790 gene. Primers used for fragment A amplification (A_Fw and A_Re) and sequencing (C_Re and D_Fw) are highlighted. The region of amplicon A corresponding to amine oxidase encoding transcript (AK357034.1 and AK363738.1) is marked with blue, the region corresponding to GSK3 encoding transcript (AK360683.1 and AK358344.1) is marked with green. The amplicon A region with no similarity to any known barley cDNA or barley EST is marked with black.
(DOCX)
