# Supplementary figures and images for: Annotation and profiling of barley GLYCOGEN SYNTHASE3/Shaggy-like genes indicated shift in organ-preferential expression
Source: PLoS One. 2018 Jun 19;13(6):e0199364. doi: 10.1371/journal.pone.0199364 (PMC6007836; doi:10.1371/journal.pone.0199364)

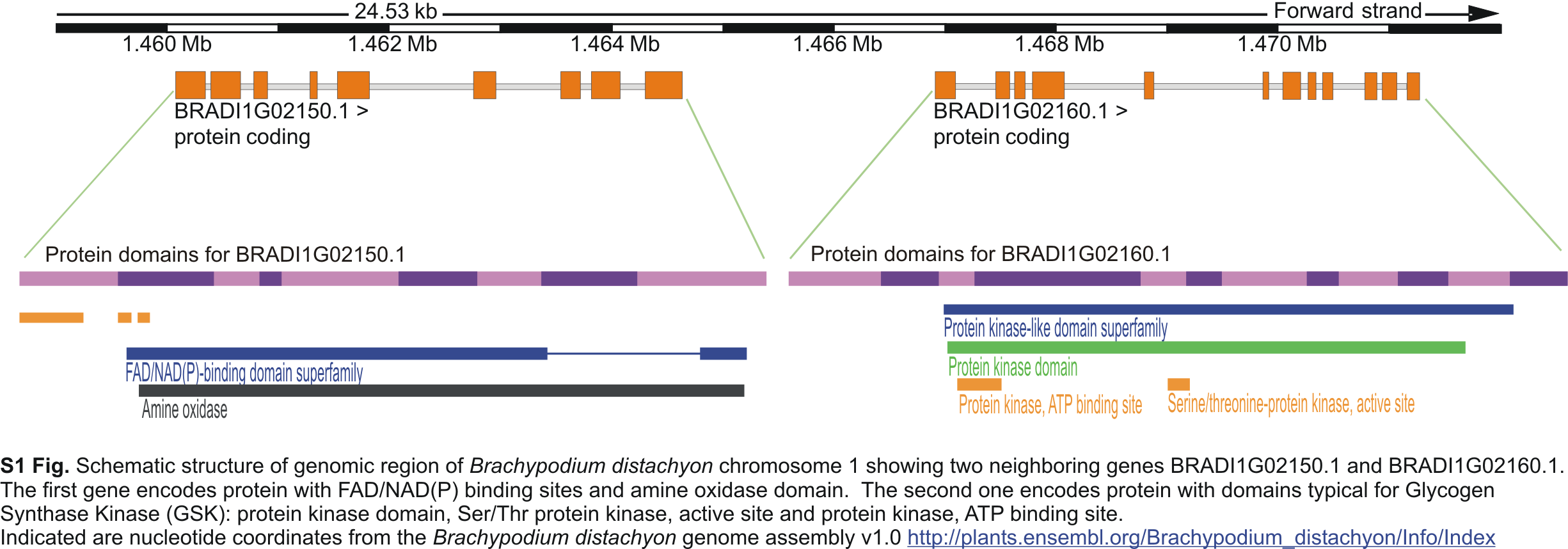

Supplement: S1 Fig — (TIF) [file pone.0199364.s001.tif]
