# Supplementary material for: Annotation and profiling of barley GLYCOGEN SYNTHASE3/Shaggy-like genes indicated shift in organ-preferential expression
Source: PLoS One. 2018 Jun 19;13(6):e0199364. doi: 10.1371/journal.pone.0199364 (PMC6007836; doi:10.1371/journal.pone.0199364)
Supplement: S1 Table — (DOCX) [file pone.0199364.s002.docx]

S1 Table. List of primers and reaction conditions used in this study.

| **Gene** | **Ensemble Plants**  **Ensamble Plants Archive*** | **NCBI** | **Primer symbol and sequence** | | **Amplicon size and reaction conditions** |
| --- | --- | --- | --- | --- | --- |
| *HvGSK1.1* | HORVU3Hr1G034440.2 | AK251287.1 | AK251_F1  AK251_R1 | CCCTTCTTTGATGAGCTTCG  CAGGGGAAATGCTCACTTGT | 221bp, 95ºC 15 min, 45 cycles (95ºC 25s, 62ºC 25s, 72ºC 25s) 72ºC 5min |
| *HvGSK1.2* | HORVU5Hr1G117030.1 | AK368391 | AK368_FR  AK368_Rev | TCTGGGCACACCTACAAGGG  TGGAGACCAGGTCCACTGCT | 139 bp, 95ºC 15 min, 45 cycles (95ºC 25s, 60ºC 25s, 72ºC 25s) 72ºC 5min |
| *HvGSK1.3* | HORVU1Hr1G016490.9 | MLOC_11953.1 | Mloc_11953_F1  Mloc_11953_R1 | ACGAGATGGGCAATATGAG  GTTCCAAATGACCCATGACC | 178 bp, 95ºC 15 min, 45 cycles (95ºC 25s, 62ºC 25s, 72ºC 25s) 72ºC 5min |
| *HvGSK2.1* | HORVU3Hr1G026020.1 | AK364823.1 | 403AK364_FR  524AK364_Re | AGTGCTTGGAGACTGGAGAGAC  GTGCTTCAGAGAGACGACATTG | 122 bp, 95ºC 15 min, 45 cycles (95ºC 25s, 60ºC 25s, 72ºC 25s) 72ºC 5min |
| *HvGSK2.2* | MLOC_68311.2* | none | Mloc_68311_F1  Mloc_68311_R1 | CACCAACTCGGGAGGAAATA  GCTCCCGTAGCTCATCAAAG | 213 bp, 95ºC 15 min, 45 cycles (95ºC 25s, 62ºC 25s, 72ºC 25s) 72ºC 5min |
| *HvGSK3.1* | HORVU1Hr1G048580.7 | AK362547.1 | 1270AK362_Fr  1393AK362_Rev | AAAGTGGCGTTGATCAGTTGG  CAGGGATGAGCTTTTATCTGAGG | 123 bp, 95ºC 15 min, 45 cycles (95ºC 25s, 60ºC 25s, 72ºC 25s) 72ºC 5min |
| *HvGSK4.1* | HORVU5Hr1G119790.18 | AK358344.1 | AK358_FR  AK358_Rev | GCGAGAAGGCAGAACCTGTT  TGTCACCCACCCACACAAAG | 133 bp, 95ºC 15 min, 45 cycles (95ºC 25s, 60ºC 25s, 72ºC 25s) 72ºC 5min |
| ADP-rybosylation factor | | AJ508228 | Ref2_FR  Ref2_Rev | GCTCTCCAACAACATTGCCAAC  GCTTCTGCCTGTCACATACGC | 162 bp, 95ºC 15 min, 45 cycles (95ºC 25s, 60ºC 25s, 72ºC 25s) 72ºC 5min |
| HORVU5Hr1G119790.1 | | | A_FW  A_Re | AAGCAGAGCTGTGGAGAACC  GAACTCAACTGAGCTGGCCT | 1719 bp, 98ºC 30 s, 36 cycles (98ºC 10 s, 66ºC 30 s, 72ºC 2 min) 72ºC 8 min |
|  |  |  | B_FW  B_Re | CACATGTTGTGCTAAAAGTG  GTCAGAGCCGGCATTATTCAGT | 806 bp, 95ºC 15 min, 36 cycles (95ºC 20s, 60ºC 40s, 72ºC 40s) 72ºC 5min |
|  |  |  | C_FW  C_Re | AGAGCTGTGGAGAACCATGC  CGCGATTCTTCTCTCCTCCC | 1024 bp, 98ºC 30 s, 36 cycles (98ºC 10 s, 64ºC 30 s, 72ºC 2 min) 72ºC 8 min |
|  |  |  | D_FW  D_Re | GCACTGAATAATGCCGGCTC  GGAGATCACCAAGGTCAGCC | 1053 bp, 98ºC 30 s, 36 cycles (98ºC 10 s, 66ºC 30 s, 72ºC 2 min) 72ºC 8 min |
