# Supplementary material for: Annotation and profiling of barley GLYCOGEN SYNTHASE3/Shaggy-like genes indicated shift in organ-preferential expression
Source: PLoS One. 2018 Jun 19;13(6):e0199364. doi: 10.1371/journal.pone.0199364 (PMC6007836; doi:10.1371/journal.pone.0199364)
Supplement: S3 Table — Boundaries of the kinase domain (Pfam AC: PF00069) are indicated by black arrows and framed in black. Alignment includes one protein isoform per gene (isoform containing kinase domain with the highest Pfam score). (DOCX) [file pone.0199364.s004.docx]

**S3 Table.** Amino acid alignment of GSK-encoded proteins in *Arabidopsis thaliana*, barley and *Physcomitrella patens.*

Boundaries of the kinase domain (Pfam AC: PF00069) are indicated by black arrows and framed in black. Alignment includes one protein isoform per gene (isoform containing kinase domain with the highest Pfam score). Other highlighted domains (description in the text): **ATP**-binding box, **CDFGSAK** and **GEPNISYICSR** motifs, **SIDIW** motif, **TREE** motif and **MEYV / LEYF** motifs. **Arg96**, **Arg180** (R) and **Lys205** (K) residues corresponding to human GSK3β and defining the pocket for primed substrate binding are marked with pink font and arrowhead. **Tyr** (Y) corresponding **Tyr 200** present within the **GEPNISYICSR** motif in BIN2, and human GSK3α and GSKβ is highlighted and marked with blue arrow.

AtSK11/ASK_alfa ------MASVG-------------------------------------------------

AtSK12/ASK_gamma ------MASVG-------------------------------------------------

AtSK13/ASK_episolon ------MASVG-------------------------------------------------

AtSK21/ASK_eta/BIN2 ------------------------------------------------------------

AtSK22/ASK_iota/BIL1 ------MASLPLG-----------------------------------------------

AtSK23/ASK_zeta/BIL2 ------MTSIPLG-----------------------------------------------

AtSK31/ASK_theta MNVMRRLKSIASGRTSISSDPGGDYALKRAKLDQENDNLCV--------DPMQVDQNSSC

AtSK32/ASK_beta MNVVRRLTSIASGRNFVSSDNVGETETPRSKPNQN-------------------------

AtSK41/ASK_kappa ------MA----------------------------------------------------

AtSK42/ASK_delta -------M----------------------------------------------------

HvGSK1.1 ------MASVG-------------------------------------------------

HvGSK1.2 ------MTSFG-------------------------------------------------

HvGSK1.3 ------MASVG-------------------------------------------------

HvGSK2.1 ------LL----------------------------------------------------

HvGSK2.2 ------MEHPA-------------------------------------------------

HvGSK3.1 MHMMRRLKSIASGRSSV-SDPGGDSGSKRPKFDQDGLGDIVIQPHLSDDKPMHLDQES--

HvGSK4.1 ------MAFSG-------------------------------------------------

Outgroup[P_patens] ------MASAT-------------------------------------------------

AtSK11/ASK_alfa ----------IAPNPGA------------RDSTGVDKLPEEMNDMKIR--------DDKE

AtSK12/ASK_gamma ----------IEPSAAV--------RESTGNVTDADRLPEEMKDMKIQ--------DDKE

AtSK13/ASK_episolon ----------TLPASSM-------ATKQSNASICAEKLPEGINEMKIK--------DDKE

AtSK21/ASK_eta/BIN2 ------------------------------------------------------MADDKE

AtSK22/ASK_iota/BIL1 ------------PQPHA-------LAP-----------PLQLHDGDALKRR-PELDSDKE

AtSK23/ASK_zeta/BIL2 -----------PPQPPS-------LAP----------QPPHLHGGDSLKRR-PDIDNDKE

AtSK31/ASK_theta FEMKADVLSQESVAGTS-------NVPAVSEKPVDDQLPDVMIEMKIRDER-NANREDKD

AtSK32/ASK_beta ----REETESTETTSYE--------KDSVSSSENSDHLPKEIREVGLG--------DDKD

AtSK41/ASK_kappa ----SSGLGNGVGTSRS-------AKGLKSSSSSVDWLTRDLAETRIRDK--VETDDERD

AtSK42/ASK_delta ----ESHLGNGVGSSRS-------AKNTKNTSSSVDWLSRDMLEMKIRDK--TEADEERD

HvGSK1.1 ----------VAPSGHK---------NSSGTSMGVEKLPDQMNDLKIR--------DDKE

HvGSK1.2 ----------VAPASGL---------RDAGGSSEVDRLPDEISNMRIS--------DEKE

HvGSK1.3 ---------AVRPSSRF--------QNDTSTSGDAERLPNEMGNMSIR--------DDRD

HvGSK2.1 -------------PSGE-------------------------------------------

HvGSK2.2 ----------PAPEPMLL----------------DEQPPTAV----------ACEKKQQD

HvGSK3.1 ----SSHRDAEASTSTS-------MNPAKAEDTGAD-LPKGMHDMTINDNKVDGHNNDKE

HvGSK4.1 ----QRHVGAAGSSSRQ-------GNGFKGQASSVEFLGRGMVGMQLRDAKPDADDDERD

Outgroup[P_patens] ----------AAAVSGTNMIGGGRAAPTKAGSSGVELLPKEMHEMKLRDDK-VDHGDDKE

▼

AtSK11/ASK_alfa MEATVVDGNGTETGHIIVTTIGGRNGQPK-QTISYMAERVVGHGSFGVVFQAKCLETGET

AtSK12/ASK_gamma MEATIVNGNVTETGHIIVTTIGGRNGQPK-QTISYMAERVVGHGSFGVVFQAKCLETGET

AtSK13/ASK_episolon MEAAVVDGNGTETGHIIVTTIGGKNGQPK-QTISYMAERIVGQGSFGIVFQAKCLETGET

AtSK21/ASK_eta/BIN2 MPAAVVDGHDQVTGHIISTTIGGKNGEPK-QTISYMAERVVGTGSFGIVFQAKCLETGET

AtSK22/ASK_iota/BIL1 MSAAVIEGNDAVTGHIISTTIGGKNGEPK-QTISYMAERVVGTGSFGIVFQAKCLETGES

AtSK23/ASK_zeta/BIL2 MSAAVIEGNDAVTGHIISTTIGGKNGEPK-QTISYMAERVVGTGSFGIVFQAKCLETGES

AtSK31/ASK_theta METTVVNGSGTETGQVITTTVGGRDGKPK-QTISYMAQRVVGTGSFGVVFQAKCLETGEQ

AtSK32/ASK_beta MDCGIIKGNGTESGRIITTKKKGLNDQKD-KTISYRAEHVIGTGSFGVVFQAKCLETEEK

AtSK41/ASK_kappa SEPDIIDGAGAEPGHVIRTTLRGRNGQSR-QTVSYISEHVVGTGSFGMVFQAKCRETGEV

AtSK42/ASK_delta SEPDIIDGVGAEPGHVITTTLPGRNGQSR-QTVSYIAEHVVGTGSFGMVFQAKCRETGEV

HvGSK1.1 VEATIINGKGTETGHIIVTTTGGKNGQPK-QTVSYMAERIVGQGSFGIVFQAKCLETGET

HvGSK1.2 VEATIINGNGTEAGHIIVTTIGGRDGQRK-QTISYMAERVIGQGSFGVVFQAKCLETSET

HvGSK1.3 PEDIVVNGNGTEPGHIIVTSIEGRNGQAK-QTISYMAERVVGHGSFGTVFQAKCLETGET

HvGSK2.1 ------DGGDWVG--LVAVRPGNHRDAAD-FTISYMAERVVGTGSFGIVFQAKCLETGET

HvGSK2.2 GEAPYAEGNDAMTGHIISTTIGGKNGEPK-QTISYMAERVVGTGSFGIVFQAKCLETGET

HvGSK3.1 AEGIIVDGNGTEAGQIIVTTIGGQNGKPKQQKVSYMAERVVGTGSFGVVYQAKCLETGET

HvGSK4.1 NEPDVVADSGAEAGQIIATTIRGRNGLPKQQSISYIAEHVVGTGSFGVVFQAKCRETGEV

Outgroup[P_patens] IEATVVDGNGTETGHIIATTIGGRNGQPK-QTISYSAERVVGTGSFGIVFQAKCIESGET

. :: . . . .:** :::::* **** *:**** *: *

AtSK11/ASK_alfa VAIKKVLQDRRYKNRELQTMRLLDHPNVVSLKHCFFSTTEKDELYLNLV**LEYV**PETVHRV

AtSK12/ASK_gamma VAIKKVLQDRRYKNRELQTMRLLDHPNVVSLKHCFFSTTEKDELYLNLV**LEYV**PETVHRV

AtSK13/ASK_episolon VAIKKVLQDKRYKNRELQTMRLLDHPNVVSLKHCFFSTTEKDELYLNLV**LEYV**PETVYRV

AtSK21/ASK_eta/BIN2 VAIKKVLQDRRYKNRELQLMRVMDHPNVVCLKHCFFSTTSKDELFLNLV**MEYV**PESLYRV

AtSK22/ASK_iota/BIL1 VAIKKVLQDRRYKNRELQLMRPMDHPNVISLKHCFFSTTSRDELFLNLV**MEYV**PETLYRV

AtSK23/ASK_zeta/BIL2 VAIKKVLQDRRYKNRELQLMRLMDHPNVVSLKHCFFSTTTRDELFLNLV**MEYV**PETLYRV

AtSK31/ASK_theta VAIKKVLQDKRYKNRELQIMRLQDHPNVVRLRHSFFSTTDKDELYLNLV**LEYV**PETVYRA

AtSK32/ASK_beta VAIKKVLQDKRYKNRELQIMRMLDHPNVVELKHSFFSTTEKDELYLNLV**LEYV**PETIYRA

AtSK41/ASK_kappa VAIKKVLQDKRYKNRELQIMQMLDHPNAVALKHSFFSRTDNEEVYLNLV**LEFV**PETVNRV

AtSK42/ASK_delta VAIKKVLQDKRYKNRELQIMQMLDHPNVVCLKHSFYSRTENEEVYLNLV**LEFV**PETVNRT

HvGSK1.1 VAIKKVLQDKRYKNRELQTMRLLDHPNVVALKHCFFSTTEKDELYLNLV**LEYV**PETVHRV

HvGSK1.2 VAIKKVLQDKRYKNRELQMMRLLDHPNVVSLKHCFFSTTEKDELFLNLV**LEYV**PETVHRV

HvGSK1.3 VAIKKVLQDKRYKNRELQTMRVLDHPNVVALKHCFFSKTEKEELYLNLV**LEYV**PETAHRV

HvGSK2.1 VAIKKVLQDRRYKNRELQLMRSMDHPNVVSLKHCFFSTTSRDELFLNLV**MEYV**PETLYRV

HvGSK2.2 VAIKKVLQDRRYKNRELQLMRSMNHSNVVSLKHCFFSTTSRDELFLNLV**MEYV**PETLYRV

HvGSK3.1 VAIKKVLQDKRYKNRELQTMQLLDHPNVVQLKHHFFSTTERGEVYLNLV**LEYV**SETVYRV

HvGSK4.1 VAIKKVLQDKRYKNRELQIMHMLDHPNIVGLKHYFFSTTEKDELYLNLV**LEFV**PETVNRM

Outgroup[P_patens] VAIKKVLQDKRYKNRELQIMRLLDHPNIVALKHCFFSTTEKDELYLNLV**LEYV**PETVYRI

*********:******** *: :*.* : *:* *:* * . *::****:*:*.*: *

AtSK11/ASK_alfa IKHYNKLNQRMPLIYVKLYTYQIFRALSYIHRCIGVCHRDIKPQNLLVNPHTHQVKLCDF

AtSK12/ASK_gamma IKHYNKLNQRMPLVYVKLYTYQIFRSLSYIHRCIGVCHRDIKPQNLLVNPHTHQVKLCDF

AtSK13/ASK_episolon SKHYSRANQRMPIIYVKLYTYQICRALAYIHGGVGVCHRDIKPQNLLVNPHTHQVKLCDF

AtSK21/ASK_eta/BIN2 LKHYSSANQRMPLVYVKLYMYQIFRGLAYIHNVAGVCHRDLKPQNLLVDPLTHQVKICDF

AtSK22/ASK_iota/BIL1 LRHYTSSNQRMPIFYVKLYTYQIFRGLAYIHTVPGVCHRDVKPQNLLVDPLTHQVKLCDF

AtSK23/ASK_zeta/BIL2 LKHYTSSNQRMPIFYVKLYTYQIFRGLAYIHTAPGVCHRDVKPQNLLVDPLTHQCKLCDF

AtSK31/ASK_theta SKHYTKMNQHMPIIFVQLYTYQICRALNYLHRVVGVCHRDIKPQNLLVNPQTHQLKICDF

AtSK32/ASK_beta SRSYTKMNQHMPLIYIQLYTYQICRAMNYLHQVVGVCHRDIKPQNLLVNNVTHEVKICDF

AtSK41/ASK_kappa ARSYSRTNQLMPLIYVKLYTYQICRALAYIHNSFGLCHRDIKPQNLLVNPHTHQLKICDF

AtSK42/ASK_delta ARSYSRMNQLMPLIYVKLYTYQICRGLAYLHNCCGLCHRDIKPQNLLVNPHTHQLKICDF

HvGSK1.1 VKHYNKMNQRMPLIYVKLYTYQICRALAYIHGSIGVCHRDIKPQNLLVNPHTHQLKLCDF

HvGSK1.2 IRHYNKMNQRMPLIYVKLYSYQICRALAYIHRTVGVCHRDIKPQNLLVNPHTHQLKICDF

HvGSK1.3 IKHYNKMNQRMPLIYAKLYMYQICRSLAYIHNSIGVCHRDIKPQNLLVNPHTHQLKLCDF

HvGSK2.1 LKHYSNANQRMPLIYVKLYMYQLFRGLAYVHTVPGVCHRDVKPQNVLVDPLTHQVKICDF

HvGSK2.2 LKHYSNAKQGMPLIYVKLYTYQLFRGLAYIHTVPGVCHRDVKPQNVLVDPLTHQVKVCDF

HvGSK3.1 AKYYCRLNQRVPILYVKLYAYQMCRALAYIHRVVGVCHRDIKPQNLLVNPHTHQLKLCDF

HvGSK4.1 ARQYNRMNQKVPLIYVKLYTYQICRALAYIHNCVGICHRDIKPQNVLVNPHTHQLKICDF

Outgroup[P_patens] AKHYNRMNQRMPLVYVKLYTYQICRSLAYIHSGIGVCHRDIKPQNLLVNPHTHQLKLCDF

: * :* :*:.: :** **: *.: *:* *:****:****:**: **: *:***

AtSK11/ASK_alfa GSAKVLVKGEPNISYICSRYYRAPELIFGATEYTTAIDVWSAGCVLAELLLGQPLFPGES

AtSK12/ASK_gamma GSAKVLVKGEPNISYICSRYYRAPELIFGATEYTTAIDVWSAGCVLAELLLGQPLFPGES

AtSK13/ASK_episolon GSAKVLVKGEPNISYICSRYYRAPELIFGATEYTTTIDIWSAGCVLAELLLGQPLFPGES

AtSK21/ASK_eta/BIN2 GSAKQLVKGEANISYICSRFYRAPELIFGATEYTTSIDIWSAGCVLAELLLGQPLFPGEN

AtSK22/ASK_iota/BIL1 GSAKVLVKGEPNISYICSRYYRAPELIFGATEYTASIDIWSAGCVLAELLLGQPLFPGEN

AtSK23/ASK_zeta/BIL2 GSAKVLVKGEANISYICSRYYRAPELIFGATEYTSSIDIWSAGCVLAELLLGQPLFPGEN

AtSK31/ASK_theta GSAKMLVPGEPNISYICSRYYRAPELIFGATEYTNAIDMWSGGCVMAELLLGQPLFPGES

AtSK32/ASK_beta GSAKMLIPGEPNISYICSRYYRAPELIFGATEYTSAIDMWSVGCVMAELFLGHPLFPGET

AtSK41/ASK_kappa GSAKVLVKGEPNVSYICSRYYRAPELIFGASEYTTAIDIWSTGCVMAELLLGQPLFPGES

AtSK42/ASK_delta GSAKVLVKGEPNISYICSRYYRAPELIFGATEYTTAIDIWSTGCVMAELLLGQPLFPGES

HvGSK1.1 GSAKVLVKGEPNISYICSRYYRAPELIFGATEYTTAIDIWSAGCVLAELMLGQPLFPGES

HvGSK1.2 GSAKVLVKGEPNISYICSRYYRAPELIFGATEYTTAIDIWSAGCVLAELLTGQPLFPGES

HvGSK1.3 GSAKVLVKGEPNISYICSRYYRAPELIFGATEYTTAIDVWSAGCVLAELLLGQPIFPGDS

HvGSK2.1 GSAKVLVPGEPNIAYICSRYYRAPELIFGATEYTTSIDIWSAGCVLAELLLGQPLFPGET

HvGSK2.2 GSAKVLVAGEPNISYICSRYYRAPELIFGATEYTSSIDIWSAGCVLAELLLGQPLFPGES

HvGSK3.1 GSAKKLVPGEPNISYICSRYYRAPELIFGATEYTTAIDIWSVGCVVAELLIGQPLFPGES

HvGSK4.1 GSAKILVKGEPNISYICSRYYRAPELIFGATEYTTAIDLWSTGCVMAELLLGQPIFPGES

Outgroup[P_patens] GSAKVLVKGEPNISYICSRYYRAPELIFGATEYTTAIDIWSMGCVMAELLLGQPLFPGES

**** *: **.*::*****:**********:*** :**:** ***:***: *:*:***:.

AtSK11/ASK_alfa GVDQLVEIIKVLGTPTREEIKCMNPNYTEFKFPQIKAHPWHKIFHKRMPPEAVDLVSRLL

AtSK12/ASK_gamma GVDQLVEIIKVLGTPTREEIKCMNPNYTEFKFPQIKAHPWHKIFHKRMPPEAVDLVSRLL

AtSK13/ASK_episolon GVDQLVEIIKVLGTPTREEIKCMNPNYTEFKFPQIKAHPWHKIFHKRTPPEAVDLVSRLL

AtSK21/ASK_eta/BIN2 AVDQLVEIIKVLGTPTREEIRCMNPHYTDFRFPQIKAHPWHKIFHKRMPPEAIDFASRLL

AtSK22/ASK_iota/BIL1 SVDQLVEIIKVLGTPTREEIRCMNPNYTDFRFPQIKAHPWHKVFHKRMPPEAIDLASRLL

AtSK23/ASK_zeta/BIL2 SVDQLVEIIKVLGTPTREEIRCMNPNYTDFRFPQIKAHPWHKVFHKRMPPEAIDLASRLL

AtSK31/ASK_theta GIDQLVEIIKILGTPTREEIRCMNPNYTEFKFPQIKAHPWHKIFHKRMPPEAVDLVSRLL

AtSK32/ASK_beta SVDQLVEIIKILGTPAREEIKNMNPRYNDFKFPQIKAQPWHKIFRRQVSPEAMDLASRLL

AtSK41/ASK_kappa GVDQLVEIIKVLGTPTREEIKCMNPNYTEFKFPQIKPHPWHKVFQKRLPPEAVDLLCRFF

AtSK42/ASK_delta GVDQLVEIIKVLGTPTREEIKCMNPNYTEFKFPQIKPHPWHKVFQKRLPPEAVDLLCRFF

HvGSK1.1 GVDQLVEIIKVLGTPTREEIKCMNPNYTEFKFPQIKAHPWHKVFHKRMPPEAVDLVSRLL

HvGSK1.2 GVDQLVEIIKILGTPTREEIKCMNPNYTEFKFPQIKAHPWHKVFHKRMPPEAVDLVSRLL

HvGSK1.3 GVDQLVEIIKVLGTPTREEIKCMNPNYTEFKFPQIKAHPWHKIFHKRMPAEAVDLVSRLL

HvGSK2.1 AVDQLVEIIKVLGTPTREEIRCMNPNYTEFRFPQIKAHPWHKIFHKRMPAEAIDLASRLL

HvGSK2.2 AVDQLVEIIKVLGTPTREEIRCMNPNYTEFRFPQIKAHPWHKVFHKKMPPEAIDLASRLL

HvGSK3.1 GVDQLVEIIKILGTPTREEIRCMNPNYSEFKFPQIKAHPWHKLFGKRMPPEAVDLVSRLL

HvGSK4.1 GVDQLVEIIKVLGTPTREEIKCMNPNYTEFKFPQIKAHPWHKVFQKKHPPEAMDLVSRFL

Outgroup[P_patens] GVDQLVEIIKVLGTPTREEIKCMNPNYTEFKFPQIKAHPWHKVFHKRMPSEAVDLVSRLL

.:********:****:****: ***.*.:*:*****.:****:* :: ..**:*: .*::

▼

AtSK11/ASK_alfa QYSPNLRSAALDTLVHPFFDELRDPNARLPNGRFLPPLFNFKPHELKGVPLEMVAKLVPE

AtSK12/ASK_gamma QYSPNLRCAALDSLVHPFFDELRDPNARLPNGRFLPPLFNFKPHELKGVPVEMVAKLVPE

AtSK13/ASK_episolon QYSPNLRSTAMEAIVHPFFDELRDPNTRLPNGRALPPLFNFKPQELKGASLELLSKLIPD

AtSK21/ASK_eta/BIN2 QYSPSLRCTALEACAHPFFDELREPNARLPNGRPFPPLFNFK-QEVAGSSPELVNKLIPD

AtSK22/ASK_iota/BIL1 QYSPSLRCTALEACAHPFFNELREPNARLPNGRPLPPLFNFK-QELGGASMELINRLIPE

AtSK23/ASK_zeta/BIL2 QYSPSLRCTALEACAHPFFNELREPNARLPNGRPLPPLFNFK-QELSGASPELINRLIPE

AtSK31/ASK_theta QYSPNLRCTALEACAHPFFDDLRDPNVSLPNGRALPPLFNFTAQELAGASTELRQRLIPA

AtSK32/ASK_beta QYSPNLRCTALEACAHPFFDDLRDPRASLPNGRALPPLFDFTAQELAGASVELRHRLIPE

AtSK41/ASK_kappa QYSPNLRCTALEACIHPLFDELRDPNTRLPNGRPLPPLFNFKPQELSGIPPEIVNRLVPE

AtSK42/ASK_delta QYSPNLRCTAVEACIHPFFDELRDPNARLPNGRPLPPLFNFKPQELSGIPPETVDRLVPE

HvGSK1.1 QYSPNLRCTAVEALVHPFFDELRDPGTRLPNGRFLPPLFNFKPNELKGIPVDVAAKLIPE

HvGSK1.2 QYSPHLRSSALDALIHPFFDELRDPNTRLPNGRFLPPLFNFKPHELKGLPMEIAMKLVPE

HvGSK1.3 QYSPSLRSTALEALIHPFFDELRDPNTRLPNGRFLPPLFNFKPHELKGVPMDILVKLIPE

HvGSK2.1 QYSPNLRCTALDACAHSFFDELREPNARLPNGRPFPPLFNFK-PELANASPELINRLVPE

HvGSK2.2 QYSPSLRCTALDACAHPFFDELREPNAHLPNGRPFPPLFNFK-HELANVSQDLINRLVPE

HvGSK3.1 QYSPNLRCTAVDACAHPFFDELRDPKTCLPNGRPLPPLFNFTGAELEGLPVELLHRIIPE

HvGSK4.1 QYSPDLRCTAMEACMHPFFDELRDPNTRLPNGRPLPPLFNFRTQELNGIPP---------

Outgroup[P_patens] QYSPNLRCNALEACVHPFFDELRDLNCRLPNGRPLPPLFNFKPQELKGATPEILQRLIPE

**** **. *::: *.:*::**: ***** :****:* *: . .

AtSK11/ASK_alfa HAKQCPWLGL-------------------------

AtSK12/ASK_gamma HARKQCPWLSL-------------------------

AtSK13/ASK_episolon HARKQCSFLAL-------------------------

AtSK21/ASK_eta/BIN2 HIKRQLGLSFLNQSGT--------------------

AtSK22/ASK_iota/BIL1 HVRRQMSTGLQNS-----------------------

AtSK23/ASK_zeta/BIL2 HVRRQMNGGFPFQAGP--------------------

AtSK31/ASK_theta HCQGTGSSS---------------------------

AtSK32/ASK_beta HARK--------------------------------

AtSK41/ASK_kappa HARKQNLFMALHS-----------------------

AtSK42/ASK_delta HARKQNHFMALHSCLRIPCREVRMRKIQHYFVRYMI

HvGSK1.1 HARKQSSHA---------------------------

HvGSK1.2 HARSQCPFLGL-------------------------

HvGSK1.3 HARKNCAFVGW-------------------------

HvGSK2.1 HVRRQNGLNFAHAGS---------------------

HvGSK2.2 HVRRQAGLAFLHAGS---------------------

HvGSK3.1 HMRK--------------------------------

HvGSK4.1 ------------------------------------

Outgroup[P_patens] HARKQNPMLAL-------------------------
