# Supplementary material for: Annotation and profiling of barley GLYCOGEN SYNTHASE3/Shaggy-like genes indicated shift in organ-preferential expression
Source: PLoS One. 2018 Jun 19;13(6):e0199364. doi: 10.1371/journal.pone.0199364 (PMC6007836; doi:10.1371/journal.pone.0199364)
Supplement: S4 Table — Primers used for fragment A amplification (A_Fw and A_Re) and sequencing (C_Re and D_Fw) are highlighted. The region of amplicon A corresponding to amine oxidase encoding transcript (AK357034.1 and AK363738.1) is marked with blue, the region corresponding to GSK3 encoding transcript (AK360683.1 and AK358344.1) is marked with green. The amplicon A region with no similarity to any known barley cDNA or barley EST is marked with black. (DOCX) [file pone.0199364.s005.docx]

**S4 Table.** Nucleotide alignment of amplicon A and the corresponding fragment of HORVU5Hr1G119790 gene.

Primers used for fragment A amplification (A_Fw and A_Re). Sequencing was primed with all indicated primers. A_Fw 5’-AAGCAGAGCTGTGGAGAACC-3’, A_Re 5’-AACTCAACTGAGCTGGCCT-3’, C_Re 5’-CGCGATTCTTCTCTCCTCCC-3’, D_Fw 5’-GCACTGAATAATGCCGGCTC-3’.

Fragment of amplicon A corresponding to amine oxidase transcript (AK357034.1 and AK363738.1) is marked with blue font, the region corresponding to GSK transcript (AK360683.1 and AK358344.1) is marked with green. The amplicon A region with no similarity to any known barley cDNA or barley EST is marked with black.

Alignment of amplicon A and HORVU5Hr1G119790 fragment shows 100% similarity of the sequences and confirms arrengement of both genes.

HORVU5Hr1G119790 frag. AAGCAGAGCTGTGGAGAACCATGCAACATTTGTGTTCAATATAAAGATGCAGAGAAAAAG

Amplicon A AAGCAGAGCTGTGGAGAACCATGCAACATTTGTGTTCAATATAAAGATGCAGAGAAAAAG

************************************************************

HORVU5Hr1G119790 frag. AAAGCAGCATATTGCTCGGTACTGCACATGAGAAGAGAAGATTTTATAGTCAGCACATAC

Amplicon A AAAGCAGCATATTGCTCGGTACTGCACATGAGAAGAGAAGATTTTATAGTCAGCACATAC

************************************************************

HORVU5Hr1G119790 frag. AGAAGAGACAGGCATAGTTTTCTTATGTACCACCTCTGTATAAAAATATAAGACGTTTTC

Amplicon A AGAAGAGACAGGCATAGTTTTCTTATGTACCACCTCTGTATAAAAATATAAGACGTTTTC

************************************************************

HORVU5Hr1G119790 frag. TGAGTTCTTATATTTTTATACAGAGGGGGTAGTTTGCAAAAAGGGGAGAAGAAGAAGGAA

Amplicon A TGAGTTCTTATATTTTTATACAGAGGGGGTAGTTTGCAAAAAGGGGAGAAGAAGAAGGAA

************************************************************

HORVU5Hr1G119790 frag. AAAAACATGGTTCAGAATGTAGATGAATTGCTACATGCTGTTTGTACATGACTGATTGTT

Amplicon A AAAAACATGGTTCAGAATGTAGATGAATTGCTACATGCTGTTTGTACATGACTGATTGTT

************************************************************

HORVU5Hr1G119790 frag. CAGGTATAAATAATGCTACTTACGTACGTACATGACATTGAGTTCCAGCACTGAATAATG

Amplicon A CAGGTATAAATAATGCTACTTACGTACGTACATGACATTGAGTTCCAGCACTGAATAATG

************************************************************

HORVU5Hr1G119790 frag. CCGGCTCTGACAGAGTGGTCTTCTGTTTCTACCGAAAGTTGTGTGGCACCCCACCTCTGT

Amplicon A CCGGCTCTGACAGAGTGGTCTTCTGTTTCTACCGAAAGTTGTGTGGCACCCCACCTCTGT

************************************************************

HORVU5Hr1G119790 frag. CCACTTCGGATCGCAATGTGTTGGATTTTGTGTTGAATATTACTCTACTAGTAATTGGAT

Amplicon A CCACTTCGGATCGCAATGTGTTGGATTTTGTGTTGAATATTACTCTACTAGTAATTGGAT

************************************************************

HORVU5Hr1G119790 frag. GCTCTGCCTATGCGTGCAACTTTGAGAGCTTGTTTCACAGGAACACACTATAAGATAATC

Amplicon A GCTCTGCCTATGCGTGCAACTTTGAGAGCTTGTTTCACAGGAACACACTATAAGATAATC

************************************************************

HORVU5Hr1G119790 frag. CTTTCACATCACATGTCGCTCCCAGCCCCAGGTAAATAATAATATTCCCATCTTGTATTA

Contig_1.seq CTTTCACATCACATGTCGCTCCCAGCCCCAGGTAAATAATAATATTCCCATCTTGTATTA

************************************************************

HORVU5Hr1G119790 frag. TCCATCCATCAGTCAATCCACAAAAATCTCGCTGTTAAATCGACTGCAAACAAAGGTGTC

Amplicon A TCCATCCATCAGTCAATCCACAAAAATCTCGCTGTTAAATCGACTGCAAACAAAGGTGTC

************************************************************

HORVU5Hr1G119790 frag. ACCTCACCTCACCTTCACAGTTCTTCAGAGTCTCAGCCCTATGCCCCGGCTTGTTTGTTT

Amplicon A ACCTCACCTCACCTTCACAGTTCTTCAGAGTCTCAGCCCTATGCCCCGGCTTGTTTGTTT

************************************************************

HORVU5Hr1G119790 frag. CCTTCTTCCTTCCGGTTCACATGCTCACCATCACCTGCAGAGGCCGGAGGCTCGTTTCCT

Amplicon A CCTTCTTCCTTCCGGTTCACATGCTCACCATCACCTGCAGAGGCCGGAGGCTCGTTTCCT

************************************************************

HORVU5Hr1G119790 frag. CCTTATTAAAAAAAAAATGCTCACCATCACCTAGTAGGAGTATTTTTCTACTCCATGCCA

Amplicon A CCTTATTAAAAAAAAAATGCTCACCATCACCTAGTAGGAGTATTTTTCTACTCCATGCCA

************************************************************

HORVU5Hr1G119790 frag. ATAATTTTCTCCAGAAAAAGTAAAGTTTGGATGGGAGAAGGGGAGCTAGCAGCCGCACTC

Amplicon A ATAATTTTCTCCAGAAAAAGTAAAGTTTGGATGGGAGAAGGGGAGCTAGCAGCCGCACTC

************************************************************

HORVU5Hr1G119790.1 TGCCCTCCACTCACCCTCACTCACTCACTCACTCTTCACAGGTCACCATTACCCACTTGC

Amplicon A TGCCCTCCACTCACCCTCACTCACTCACTCACTCTTCACAGGTCACCATTACCCACTTGC

************************************************************

HORVU5Hr1G119790 frag. TCCTGAGACCGCCGGCCGGCCCTCACCGTCTCCCTCCGCCGGCGCACCGAACTCGGCAGT

Amplicon A TCCTGAGACCGCCGGCCGGCCCTCACCGTCTCCCTCCGCCGGCGCACCGAACTCGGCAGT

************************************************************

HORVU5Hr1G119790 frag. CCCTGTAGGGAGGAGAGAAGAATCGCGCCTCCCTTGATCTGCTCCCAGAAATTGCGGCGA

Amplicon A CCCTGTAGGGAGGAGAGAAGAATCGCGCCTCCCTTGATCTGCTCCCAGAAATTGCGGCGA

************************************************************

HORVU5Hr1G119790 frag. AGGTACCGGCTCTTGGATTCTTTGATCCCACAAAAGAAAAAGTTCATTTCTTTAATGCTC

Amplicon A AGGTACCGGCTCTTGGATTCTTTGATCCCACAAAAGAAAAAGTTCATTTCTTTAATGCTC

************************************************************

HORVU5Hr1G119790 frag. GCGCCGGCGCCTGTCTGCGGCCGTTTGATTCGGGAGGGATGGATTGTCGGGTTTATCTCC

Amplicon A GCGCCGGCGCCTGTCTGCGGCCGTTTGATTCGGGAGGGATGGATTGTCGGGTTTATCTCC

************************************************************

HORVU5Hr1G119790 frag. CAGGATCTTAGCCCAATTTCGAGATTGGCCCCTCGCGATTTGGGGAGAGCCGTTTTTATT

Amplicon A CAGGATCTTAGCCCAATTTCGAGATTGGCCCCTCGCGATTTGGGGAGAGCCGTTTTTATT

************************************************************

HORVU5Hr1G119790 frag. CATCTGTTTATTTATTTATTTATTCGGTGGATCTTCTCGGTCTCTGGGTGGGTGGGTAGA

Amplicon A CATCTGTTTATTTATTTATTTATTCGGTGGATCTTCTCGGTCTCTGGGTGGGTGGGTAGA

************************************************************

HORVU5Hr1G119790 frag. GGTTCTGCTCATGGATTTTGTGGAGATGTGTGCTCTGACAGTCCGACTGAAGAATTATCA

Amplicon A GGTTCTGCTCATGGATTTTGTGGAGATGTGTGCTCTGACAGTCCGACTGAAGAATTATCA

************************************************************

HORVU5Hr1G119790 frag. GTTGTCCGCAAATTTAAGAACTGGAGATTTTCCCCAATCAAAGTTACCAATCTGAGGTCT

Amplicon A GTTGTCCGCAAATTTAAGAACTGGAGATTTTCCCCAATCAAAGTTACCAATCTGAGGTCT

************************************************************

HORVU5Hr1G119790 frag. TGTACTGAATTTTTCTTAAACTGTTTTTTGGGTGGGATGTTGATGCAGGTCTTGTTGAAG

Amplicon A TGTACTGAATTTTTCTTAAACTGTTTTTTGGGTGGGATGTTGATGCAGGTCTTGTTGAAG

************************************************************

HORVU5Hr1G119790 frag. CAGGTGGTGGAGGAAGCTGCGTTGGCAGTGGCAGGCTCATCCCTCTGCTGGCAATGAGAT

Amplicon A CAGGTGGTGGAGGAAGCTGCGTTGGCAGTGGCAGGCTCATCCCTCTGCTGGCAATGAGAT

************************************************************

HORVU5Hr1G119790 frag. GAGCCCCCTGCTGTTTGCAGAGGCTGACCTTGGTGATCTCCATTGATCCCTGCCAATCAG

Amplicon A GAGCCCCCTGCTGTTTGCAGAGGCTGACCTTGGTGATCTCCATTGATCCCTGCCAATCAG

************************************************************

HORVU5Hr1G119790 frag. CTGGGAGATGGCCTTTTCCGGACAGAGGCATGTCGGTGCCGCCGGGAGCTCGTCGCGGCA

Amplicon A CTGGGAGATGGCCTTTTCCGGACAGAGGCATGTCGGTGCCGCCGGGAGCTCGTCGCGGCA

************************************************************

HORVU5Hr1G119790 frag. GGGGAATGGCTTCAAGGGGCAGGCCAGCTCAGTTGAGTTC

Amplicon A GGGGAATGGCTTCAAGGGGCAGGCCAGCTCAGTTGAGTTC

****************************************
